# The procognitive effects of leptin in the brain and their clinical implications

**DOI:** 10.1111/j.1742-1241.2010.02536.x

**Published:** 2010-12

**Authors:** G Paz-Filho, M-L Wong, J Licinio

**Affiliations:** Department of Translational Medicine, John Curtin School of Medical Research, The Australian National UniversityCanberra, Australia

## Abstract

**Background:**

Leptin is a pleiotropic hormone produced mainly by the adipose tissue. Its most well-known effect is to regulate food intake and energy metabolism within the hypothalamus. More recently, several peripheral and extra-hypothalamic effects have been described, expanding leptin's actions far beyond energy balance.

**Aims:**

To review the extra-hypothalamic effects of leptin and their possible clinical implications.

**Methods:**

We did a PubMed search using the terms “leptin” AND “brain” AND “neuron” AND “glial”, and selected the most relevant articles.

**Results:**

In extra-hypothalamic sites, leptin has remarkable effects on neurogenesis, axon growth, synaptogenesis, denditric morphology, development of oligodendroglial cells, neuron excitability, neuroprotection and regulation of beta-amyloid levels. Those effects have been shown to improve cognition and mood in animal models of depression and anxiety. In lean humans, leptin levels have been negatively correlated with the development of Alzheimer’s disease.

**Conclusions:**

Leptin has extra-hypothalamic effects that may protect the brain against the development of mood and neurodegenerative disorders, such as Alzheimer’s disease. Better understanding of those effects may lead to the development of potential leptin-based therapies against such conditions.

**What’s known**

Leptin has hypothalamic and extra-hypothalamic effects. The latter include actions on neuron structure and function, as well as on glial cells. Those effects are neuroprotective and have been shown to improve cognition in animal models, as well as in a child with congenital leptin-deficiency.

**What’s new**

This article is a comprehensive compilation of extra-hypothalamic effects of leptin. We describe leptin actions on neuronal and glial structure and function, and discuss how those actions may be clinically useful for treating neurodegenerative diseases and mood disorders.

Leptin plays a crucial role as an indicator of the size of somatic energy stores (fat mass) and it is a major contributor to the regulation of energy homoeostasis (1). Its most important function is to regulate energy expenditure and food intake. As an adipokine, leptin also plays multiple functions in reproduction (2,3), glucose homoeostasis (4,5), bone formation (6,7), tissue remodelling (8), inflammation (9), as well as in other elements of the endocrine (10,11) and immune systems (12).

More recently, it has been shown that leptin has crucial extra-hypothalamic functions on brain structure and function. In animals, leptin alters structure in regions such as the midbrain (13,14), the hippocampus (15) and the hindbrain (16). Those effects of leptin on brain structure are determined by its actions on neurogenesis, axon growth, synaptogenesis and dendritic morphology, which occur both during pre- and postnatal life, and are important for the establishment hypothalamic, hippocampal and cortical pathways (17). In addition, several studies suggest that leptin has neuroprotective actions, by inhibiting apoptotic cell death, attenuating cell death, improving cell survival, protecting against glutamatergic cytotoxicity, protecting against oxidative stress and promoting the proliferation of hippocampal progenitor cells (18,19).

Mediated by the synaptic activation of NR2A-containing NMDA (*N*-methyl-D-aspartate) receptors and of the mitogen-activated protein kinase MAPK/ERK pathway, leptin regulates the synapse morphology of hippocampal neurons, enhancing the motility and density of dendritic filopodia (20). Furthermore, leptin is a potent neurogenic factor not only to hipoccampal (21,22), but also to cortical neurons (23). The effects of leptin on axonal growth are not restricted to the hippocampus, and are also evident in the cortex (24). Leptin has additional effects on the development of oligodendroglial cells (25), which may contribute to the structural and functional changes determined by the adipokine. Furthermore, effects of leptin on brain function may be determined by its actions not only on structure, but also on neuron excitability, via the activation as well as trafficking of ATP-sensitive K^+^ channels in the hypothalamus and Ca^2+^-activated K^+^ channels in the hippocampus, and by the regulation of mesolimbic neuron excitability through yet undefined mechanisms (26,27).

In humans, leptin also has important extra-hypothalamic effects. Six and 18 months of physiological leptin replacement therapy in adults treated by our group for congenital leptin deficiency led to increases in grey matter concentration in the anterior cingulate gyrus, the inferior parietal lobule, and the cerebellum (28). More importantly, these effects were sustained over 3 years of leptin replacement and were reversed after a few weeks of leptin withdrawal (Licinio et al., unpublished results). Leptin-induced alterations of brain structure overlapped anatomically with effects on the functional magnetic resonance imaging (fMRI) response to food cues, with reductions of brain activation in regions linked to hunger (insula, parietal and temporal cortex) and enhancements of the activation of regions linked to inhibition and satiety (prefrontal cortex) ((29) and Licinio et al., unpublished results). In leptin-deficient children, these effects may be even more dramatic. In a 7-year-old leptin-deficient boy, leptin replacement, besides leading to improvement of metabolic and anthropometric parameters, also had a markedly positive effect on neurocognitive development (30).

Given these irrefutable effects on brain structure and function, it is reasonable to hypothesise that leptin may have roles on mood and cognitive disorders. Systemic and intrahippocampal infusion of leptin produced an antidepressant-like effect in mice submitted to chronic stress, through the direct activation of leptin receptors in the hippocampus (15). It has also been suggested that leptin has anxiolytic-like effects in stressed mice (31). Anorexia nervosa is another disorder that presents with low leptin levels, which directly leads not only to somatic alterations, but also to psychopathological, cognitive and sleep disorders (32). These alterations can, at least partially, be reversed by leptin treatment. Memory may also be improved by leptin as local injections of leptin into the hippocampus facilitate spatial learning, memory performance and hippocampal long-term potentiation (LTP, a form of synaptic plasticity) in mice. Leptin facilitates the conversion of short-term potentiation into LTP, by enhancing NMDA receptor function, enhances LTP at hippocampal CA1 synapses, and rapidly remodels dendrites, which explain its effects on hippocampal LTP (26,33–35).

More recently, low leptin levels have been implicated as a direct cause of cognitive impairment, particularly Alzheimer’s disease (AD). In that case, the absence of beneficial effects of leptin in the central nervous system (CNS) would predispose to cognitive impairment. A small study showed that patients with AD and vascular dementia have lower serum leptin levels (36). In a prospective study with 2,871 elderly people, those in the high leptin group had lesser likelihood of developing cognitive decline over 4 years than those in the lower group (OR = 0.66; 95% CI 0.48–0.91, after adjustment for age, race, gender, education, baseline cognitive score, hypertension, prior MI, diabetes, number of days spent in the hospital in the prior 5 years, BMI and total percentage of body fat) (37). In another prospective study of 785 healthy persons from the Framingham cohort and followed for a median of 8.3 years, 111 developed incident dementia and 89 were diagnosed with AD (38). In multivariate models, higher leptin levels were associated with a lower risk of dementia and AD (hazard ratio per 1-SD increase in log leptin was 0.68 [95% CI, 0.54–0.87] for all-cause dementia and 0.60 [95% CI, 0.46–0.79] for AD). Participants in the lowest leptin quartile were at a fourfold higher risk for developing AD in 12 years, compared with the participants in the highest quartile (25% vs. 6%). In concordance to previous studies, leptin levels were correlated with total brain volumes (28,39). However, the protective effect of leptin against the development of AD was observed only among lean individuals; and obese humans, despite having high leptin levels, may not benefit from protective effects of leptin because of central leptin resistance. Nevertheless, this is an important study further evidencing the roles of leptin on brain structure and function.

The adipoinsular axis – with leptin and insulin as its main components – has important roles on the regulation of brain function (40). Over the past decades, several studies have been proposing links among leptin, obesity, diabetes and AD. The Rotterdam study and others that followed suggested an increased risk to develop dementia and AD in patients with type 2 diabetes mellitus (41,42). In that case, insulin resistance and low insulin levels in the CNS (interestingly referred as “diabetes of the brain”) would lead to the accumulation of beta-amyloid (the pathologic hallmark of AD) and cognitive impairment. Cerebrovascular and central inflammation would contribute further to the pathogenesis of AD (43).

This state of insufficient insulin signalling and excess extracellular glucose would i) decrease astrocyte glucose uptake; ii) decrease neuronal nitric oxide synthase and increase the activity of the proinflammatory inducible nitric oxide synthase, decreasing energy substrates and oxidant supply of neurons; iii) impair redox balance by increasing peroxynitrite formation; and iv) worsen the reduced astrocyte supply and inflammation by inhibiting astrocyte AMP-activated protein kinase, which decreases glycolysis and ketogenesis and stimulates the synthesis of pro-apoptotic ceramides. Diabetes/obesity-related hyperleptinemia contributes to inhibit astrocyte 5′ adenosine monophosphate-activated protein kinase (AMPK) selectively, worsening the metabolic disruption in those cells (44).

The importance of leptin and insulin signalling on cognition and AD was further demonstrated by a study where Takeda et al. cross-bred APP23 mice (a well-studied AD mouse model) with ob/ob (leptin-deficient) and NSY mice (a lean model of type 2 diabetes). The APP+ -ob/ob and the APP+ -NSY mice had exacerbated cognitive dysfunction, with reduced brain insulin and impaired Akt phosphorylation – a key step in insulin signalling (43), and an important mediator for the proliferation of adult hippocampal neural progenitor cells (22). Furthermore, APP+ -ob/ob mice had more pronounced cognitive impairment, despite having similar insulin levels. Therefore, in the pathogenesis of AD, both leptin and insulin may play important roles, which are likely to be simultaneous and inter-dependent.

Obesity itself is a known risk factor for AD (45). Obese patients present with several brain tissue deficits, particularly in the frontal and occipital lobes, anterior cingulate gyrus, hippocampus and thalamus (46,47). Other changes found in obese patients include grey matter deficits in the frontal lobes and postcentral gyri, but enlarged volumes in the orbitofrontal white matter (48), and neuronal and myelin abnormalities in the frontal lobes (49). These changes may be consequence of several underlying factors, such as hypercortisolemia, reduced exercise, impaired respiratory function, inflammation, cardiovascular/hypertension/hyperlipidemia and type 2 diabetes (46). However, in common obesity, leptin is increased, which would theoretically be a protective factor against AD. This is not the case, as a result of the increased leptin resistance that is observed in patients with common obesity (50). Therefore, based on the lack of association between leptin levels and the development of AD in obese patients observed by Lieb et al. (38), it is likely that high leptin levels do not promote satiety nor have neuroprotective actions in those individuals.

It is now widely known that leptin has several extra-hypothalamic actions, perhaps more important than the classic effects on the regulation of food intake. Protective effects of leptin against AD may be determined not only by its actions on neuronal growth and function (Table 1), but also by its ability to regulate *in vitro* and *in vivo* beta-amyloid levels. Leptin reduces its extra-cellular levels, reduces beta-secretase activity in neuronal cells (a protease that cleaves amyloid precursor protein into beta-amyloid), increases apoE-dependent beta-amyloid uptake and increases beta-amyloid clearance from the brain to the blood by binding to megalin/LRP2 (a receptor involved in the endocytic uptake of known carriers of beta-amyloid) (51). By decreasing the accumulation of intraneuronal lipids, leptin suppresses amyloidogenic pathways. In addition, by inhibiting GSK-3β (a tau kinase), leptin reduces protein tau phosphorylation, reducing the formation of neurofibrillary tangles (another pathological hallmark of AD) (52).

**Table 1:** Possible effects of leptin in the brain that may protect against AD

Neurogenesis	
Axon growth	
Synaptogenesis	
Dendritic morphology	
Development of oligodendroglial cells	
Neuron excitability	
Neuroprotection	Inhibition/attenuation of apoptotic cell death
	Improvement of cell survival
	Protection against glutamatergic cytotoxicity
	Protection against oxidative stress
	Promotion of the proliferation of hippocampal progenitor cells
Regulation of beta-amyloid levels	Reduction of beta-amyloid extra-cellular levels
	Reduction of beta-secretase activity
	Increase of ApoE-dependent beta-amyloid uptake
	Increase of beta-amyloid clearance
	Decrease of amyloidogenic pathways
	Reduction of protein tau phosphorylation

The inhibitory effects of leptin on the formation of beta-amyloid and neurofibrillary tangles seem to be mediated by the selective activation of AMPK in neurons (53). Therefore, leptin has dual effects on AMPK: it activates the neuronal and inhibits the glial enzyme. Even among neurons, leptin can inactivate, instead of activate AMPK in certain cell types, such as hypothalamic and hippocampal neurons (54). In fact, AMPK monitors and controls cellular energy status, and seems to be the link between nutrition, cognition and survival. Low-grade hippocampal AMPK activation promotes neurogenesis and improves cognition, and high-grade hippocampal AMPK activation promotes neuronal apoptosis and cognitive impairment (55). Moderate diet restriction decreases leptin levels, without compromising its signalling. Severe diet restriction leads to a decrease in leptin levels and impairment in its signalling, and to high-grade hippocampal AMPK activation, with impairment of eight-arm maze performance. Subsequent leptin replacement in those mice reduced AMPK activation and reversed the impaired maze performance. The results of this study suggest a role for leptin as a brake to AMPK overactivity in the hippocampus to maintain neural function under energy restriction. In this case, the balance between leptin and AMPK is essential in determining neuronal proliferation or apoptosis, and cognitive performance (55).

The roles of leptin on brain structure and function are being extensively characterised by studies showing that the human brain is highly neuroplastic and depends on leptin for its proper development. Additional studies in different populations need to confirm the role of leptin as a biomarker for neurodegenerative diseases. After this role is well-established and fully characterised, leptin replacement may be regarded as a therapeutic agent in cognitive diseases, particularly in AD. However, it is first necessary to understand better its effects in the human brain, as well as in other organ systems. For example, leptin replacement increases T-cell responsiveness (56), and when in excess, it may lead to the development of autoimmune diseases (57). Leptin also stimulates growth and inhibits apoptosis of cancer cells, and may contribute to the increase in the incidence of different types of cancers that are observed in obese individuals, through the activation of JAK2-linked PI3K/Akt and MEK/ERK1/2 pathways (58).

In addition to better understanding the leptin physiology, central leptin resistance must be overcome to make leptin therapy effective in obese patients. However, in case central leptin resistance is overcome, one should be aware of effects of leptin on metabolic pathways. It is possible that exogenous leptin, in addition to having positive effects on brain structure and function, may also selectively inhibit the AMPK activity of astrocytes (the only cell types in the brain capable to perform beta oxidation of free fatty acids), leading to impaired energy metabolism and apoptosis (44). Further studies need to evaluate effects of leptin on brain structure, function and metabolism concomitantly to provide a solid foundation for work aimed at assessing possible roles of leptin in the experimental treatment of AD. Based on existing, independent lines of evidence, we believe that the hypothesis that activation of the leptin pathway may be a viable treatment strategy for AD must be tested.
